# Challenges and Solutions in the Management of Hepatocellular Carcinoma Associated with Non-Alcoholic Fatty Liver Disease

**DOI:** 10.3390/life13101987

**Published:** 2023-09-29

**Authors:** Ramona Cadar, Corina Lupascu Ursulescu, Alin Mihai Vasilescu, Ana Maria Trofin, Mihai Zabara, Delia Rusu-Andriesi, Bogdan Ciuntu, Cristina Muzica, Cristian Dumitru Lupascu

**Affiliations:** 1 Department of Surgery, Gr. T. Popa University of Medicine and Pharmacy, 700115 Iasi, Romania; ramona-petronela.cadar@umfiasi.ro (R.C.); ana-maria.trofin@umfiasi.ro (A.M.T.); mihai.zabara@umfiasi.ro (M.Z.); bogdan.ciuntu@umfiasi.ro (B.C.); cristian.lupascu@umfiasi.ro (C.D.L.); 2 General Surgery and Liver Transplant Clinic, St. Spiridon University Hospital, 700111 Iasi, Romania; 3 Department of Radiology, Gr. T. Popa University of Medicine and Pharmacy, 700115 Iasi, Romania; corina.ursulescu@umfiasi.ro; 4 Radiology Clinic, St. Spiridon University Hospital, 700111 Iasi, Romania; 5 Department of Gastroenterology, Gr. T. Popa University of Medicine and Pharmacy, 700115 Iasi, Romania; 6 Institute of Gastroenterology and Hepatology, St. Spiridon University Hospital, 700111 Iasi, Romania

**Keywords:** non-alcoholic fatty liver disease, hepatocellular carcinoma, management, challenges and solutions

## Abstract

Non-alcoholic fatty liver disease (NAFLD) has gained attention in the last few years due to its increasing prevalence worldwide becoming a global epidemic. The increasing incidence of NAFLD and the concurrent increase in the number of hepatocellular carcinoma (HCC) cases at a global level is a matter of concern. HCC has several risk factors, of which NAFLD and its associated metabolic disturbances—type 2 diabetes mellitus, obesity, and dyslipidemia—are of great interest due to their accelerating rise in incidence worldwide. There is a high amount of data derived from basic and clinical studies that reveal the molecular pathways that drive NAFLD-associated HCC. Based on these findings, new prevention, surveillance, and treatment strategies are emerging. However, current data on treatment modalities in NAFLD-associated HCC are still scarce, though the results from non-NAFLD HCC studies are promising and could provide a basis for a future research agenda to address NAFLD/NASH patients. Clinicians should carefully assess all the clinical and radiological parameters and establish a prognosis based on the Barcelona Clinic Liver Cancer classification and discuss in a multidisciplinary team the treatment strategy. The specific factors associated with NAFLD-associated HCC which can have a negative impact on survival even in patients with early HCC, such as cardiovascular disease, type 2 diabetes, and obesity, should be taken into consideration. This review aims to discuss the latest recommendations regarding the diagnosis and treatment of NAFLD-associated HCC and the remaining challenges.

## 1. Introduction

**Figure 1:**
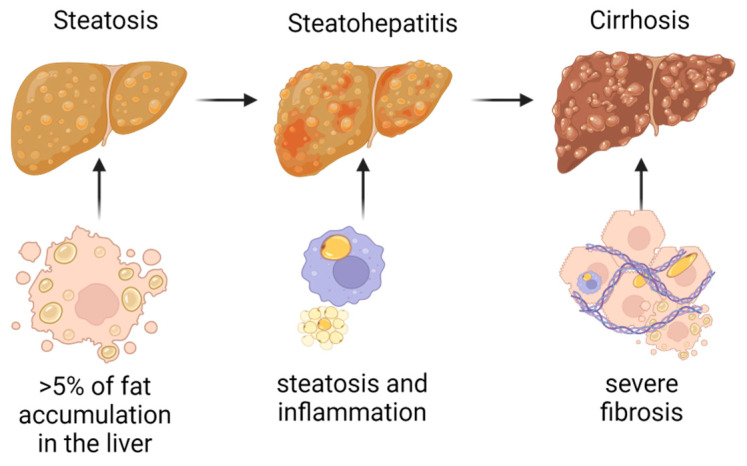
Progression from steatosis to cirrhosis.

The global prevalence of non-alcoholic fatty liver disease (NAFLD) is 25%, registering an alarming increase in recent years (from 15% in 2005 to 32% in 2023) as a consequence of the dramatic change in lifestyle that mainly includes an increased level of sedentarism and a high-fat (PUFA) and high-sucrose and -artificial sugars diet [1,2]. NAFLD occurs secondarily to the accumulation of excess triglycerides in the liver in the absence of excessive alcohol intake, which can lead to the appearance of non-alcoholic steatohepatitis (NASH) in approximately 30% of patients, which evolves in 10–20% of cases to liver cirrhosis [3,4]. Steatosis is defined as fat accumulation in liver volume more than 5%; steatohepatitis features steatosis, hepatocyte ballooning, and inflammation with rapid progression to fibrosis; and cirrhosis is defined by a diffuse hepatic process characterized by severe fibrosis and conversion of the normal liver architecture (Figure 1) [5]. The definition of NAFLD precludes alcoholic liver disease. Although there is no consensus on the threshold of alcohol consumption to exclude NAFLD, a level of 30 g/day for men and 20 g/day for women is commonly used [6]. However, light (1.0–9.9 g/day) or moderate (10.0–29.9 g/day; 10.0–19.9 g/day for women) alcohol consumption in patients with NAFLD is not uncommon [7]. The liver injury induced by alcohol encompasses structural damage to hepatocytes with lipid accumulation and inflammation. The pathways of alcohol-induced liver injury include increased regulatory activity of kinases, transcription factors, kinases, and microRNAs (miRNAs) [8].

Considering that a major aspect of establishing the diagnosis of NAFLD is to rule out alcohol consumption, which is often underestimated, in recent years, efforts have been made to change the current nomenclature of the disease, with many researchers relying on a widely accepted, inclusive name, an umbrella term to facilitate rapid diagnosis using non-invasive diagnostic techniques and therapeutic opportunities based on the control of changes in metabolic parameters. An important change has been brought at the EASL Congress in 2023, where the multinational liver society leaders from La Asociación Latinoamericana para el Estudio del Hígado (ALEH), the American Association for the Study of Liver Diseases (AASLD), and the European Association for the Study of the Liver (EASL), as well as the co-chairs of the NAFLD Nomenclature Initiative, announced that steatotic liver disease (SLD) was chosen as an umbrella term to encompass the various etiologies of steatosis [9].

However, it has been decided that steatohepatitis should be retained further as an important term in the spectrum of steatotic liver diseases. NAFLD will further metabolic dysfunction-associated steatotic liver disease (MASLD), which includes patients who have hepatic steatosis and have at least one of five cardiometabolic risk factors (central obesity, or increased waist circumference; raised triglycerides; reduced HDL cholesterol; high blood pressure; and raised fasting plasma glucose) [9]. Furthermore, the experts decided to develop a new category of patients named MetALD, which includes patients with MASLD with alcohol intake greater than that allowed for MASLD/NAFLD (140–350 g/week for females and 210–420 g/week for males) [9]. Those with no metabolic parameters and no known cause have cryptogenic SLD. Metabolic dysfunction-associated steatohepatitis (MASH) is the replacement term for non-alcoholic steatohepatitis (NASH). This new nomenclature is meant to be a common lexicon for hepatologists worldwide and should ease the diagnostic workup. Due to its recent release, the new nomenclature is soon to be implemented in clinical practice; thus, we will discuss further referring to MASLD as NAFLD.

There has been reported a concurrent increase in incidence rates of NAFLD and hepatocellular carcinoma (HCC) cases in developed countries, with most data coming from studies conducted in the USA, where 10–20% of HCC cases are attributed to NAFLD [10,11,12]. The asymptomatic clinical setting of NAFLD results in a delayed diagnosis, with negative implications for HCC surveillance strategies. Studies investigating the incidence of HCC in patients with liver cirrhosis of different etiologies reported that NAFLD cirrhosis was diagnosed much later in the course of the disease compared to the other etiologies [13,14]. NAFLD may act synergistically with chronic HCV infection or excessive alcohol consumption, leading to HCC progression [15].

Over the past few decades, liver cancer incidence and death have both been steadily increasing. With a total of 905,677 new cases reported in 2020, liver cancer constituted the sixth most prevalent cancer globally. Liver cancer still has a poor prognosis despite recent improvements. In terms of cancer-related deaths in 2020, liver cancer came in third with 830,180 fatalities [16]. HCC has several risk factors, of which NAFLD and its associated metabolic disturbances—type 2 diabetes mellitus, obesity, and dyslipidemia—are of great interest due to their accelerating rise in incidence worldwide [12].

The pathophysiology, surveillance, and treatment of NAFLD-HCC are discussed in this review based on the latest research. It also identifies current difficulties brought on by this condition and offers recommendations for how to deal with them.

## 2. From NAFLD to HCC

In most cases, the initiation, promotion, and progression of hepatocarcinogenesis take place in the presence of a microenvironment characterized by severe liver fibrosis, in which the presence of a self-perpetuating cycle of inflammation, necrosis, and fibrosis leads to the accumulation of genetic and epigenetic alterations, the dysregulation of signaling pathways, and the activation or inadequate suppression of proto-oncogenes and tumor suppressor proteins. Liver cirrhosis is a major risk factor for HCC, with a cumulative 5-year risk of 5–30%, depending on the etiological agent of the liver disease, geographic area, ethnic group, and liver cirrhosis stage [17].

It is well known that immune pathways that act as both promoters and accelerators of carcinogenesis modulate liver tumorigenesis in NAFLD. For instance, in a murine model of NASH induced by a choline-deficient high-fat diet (CDHFT), a significant acceleration of tumorigenesis occurred as a consequence of CD8+ T and natural killer T cell activation [18]. Furthermore, it seems that a high level of CD8+PD1+ T cells in the liver parenchyma has a negative impact on immune system surveillance, which consequently triggers tumorigenesis in NASH [19]. On the other hand, CD4+ T cells are key factors for efficient immune surveillance, which decreases the risk of hepatocyte malignant transformation [20]. A murine study that evaluated the effects of decreased CD4+ T cells in an MYC oncogene transgenic mouse model fed with a methionine–choline-deficient diet demonstrated that it subsequently drives HCC development [21]. Furthermore, NAFLD-driven chronic inflammation leads to the suppression of cytotoxic CD8+ T lymphocytes by IgA+ cells, thereby disrupting immune surveillance and promoting HCC development [22]. Immune checkpoint inhibitors (ICIs) are thought to restore tumor immune surveillance by targeting the programmed cell death-1 receptor (PD1; nivolumab and pembrolizumab) on exhausted CD8+ T cells or the programmed cell death-1 ligand 1 (PDL1; atezolizumab) [23,24,25,26].

Apoptosis, necroptosis, and ferroptosis are three types of programmed cell death that have been linked to the etiology of several liver illnesses. The primary morphological features of ferroptosis include cell volume reduction, a reduction in mitochondrial cristae, and an increase in mitochondrial membrane density without conventional apoptotic or necrotic manifestations [27]. Liver cells are more sensitive to ferroptosis in conditions such as liver damage, steatohepatitis, fibrosis, and cirrhosis; in contrast, liver cancer cells are either intrinsically resistant to ferroptosis or develop resistance to it [28]. Ferroptosis exhibits a dual function in the context of carcinogenesis, wherein it can either promote or restrict tumor growth. This dichotomy is contingent upon the liberation of damage-associated molecular patterns and the subsequent initiation of immune responses that are instigated by the occurrence of ferroptotic damage within the microenvironment of the tumor. In addition, it is worth noting that ferroptosis has a significant impact on the effectiveness of chemotherapy, radiation, and immunotherapy in individuals diagnosed with HCC.

## 3. Diagnosis of NAFLD-Associated HCC

Alpha-fetoprotein (AFP) is the most commonly used biomarker for the diagnosis of HCC. AFP is a 70 kD glycoprotein with a structure similar to albumin, produced in the first trimester of pregnancy by the fetal liver and yolk sac [29]. Data from recent research show that for the threshold value of 20 ng/mL at a prevalence of HCC of 5%, the positive predictive value (PPV) is 25% and the negative predictive value (NPV) is 97.7%, and at a prevalence of 20%, PPV is 61% and NPV is 90% [30]. Moreover, APASL and AASLD recommend AFP as a serologic marker for HCC to be determined semi-annually along with US, whereas EASL does not support its use due to its low cost–efficacy index [7,31,32]. Considering that HCC surveillance strategies should be based on the most available, non-invasive, and cost-efficient technique, which at the same time needs to have high sensitivity and specificity, US is the most appropriate tool recommended by all current guidelines. Regarding the performance of US for detecting HCC, a systematic review demonstrated an excellent sensitivity of 94% for any stage but a poor sensitivity of 63% for early tumors [33]. Once a nodule is identified, a more sensitive imaging method is recommended for establishing the diagnosis: CT, MRI, or contrast-enhanced ultrasound (CEUS). In a meta-analysis that compared the accuracy of MRI vs. CT in diagnosing HCC, MRI had better sensitivity and specificity than CT (82% vs. 66% and 92% vs. 91%, respectively), with significantly higher sensitivity for HCCs smaller than 1 cm (46% vs. 69%) [34]. Still, there are several shortcomings that limit the use of MRI on a daily basis in clinical practice, such as low availability, high costs, time consumption, and image quality variability. Regarding CEUS, a meta-analysis showed a sensitivity and specificity of 85% and 91%, but it should be noted that operator- and patient-related factors decrease the accuracy of CEUS, which limits its use in favor of CT or MRI [35,36]. The LiRADS criteria encompass tumor features observed on CT, MRI, and CEUS, such as tumor size, arterial hyperenhancement, wash-out, threshold growth, and capsule, and represent a common lexicon meant to increase the rates of HCC diagnosis.

According to current guidelines, the diagnostic algorithm for HCC is mainly based on the size of the nodule identified by US. Thus, a lesion of ≥10 mm on ultrasound or an AFP level >20 ng/mL should be assessed by a more sensitive method, such as CT or MRI [30,31]. Liver biopsy is reserved for the uncertain cases evaluated by both CT and MRI [30,31].

In terms of materials science and medical diagnostics, nanotechnology has emerged as a new frontier study. The goal of nanotechnology research and development has always been to combine its special qualities with medications for the imaging and treatment of HCC in order to promote its precise therapy, as opposed to standard targeting agents or chemical carriers [37]. HCC can now be diagnosed more accurately thanks to advanced ultrasound imaging technology enhanced with nanomaterials. Special prodrug nanobubbles are very useful in the early detection of liver cancer as ultrasonic contrast agents. These nanomaterials are employed in cooperative hyperthermia because they can transform ultrasonic energy into heat. For instance, NBS-GPC3-reduced graphene oxide (RGO), with its acceptable particle size, imaging capability, and photothermal efficiency, has been developed as an ultrasonic-assisted photothermal agent (37). Nanoimaging combined with CT and MRI strategies is also gaining interest based on the excellent results reported by recent studies [38,39].

After a diagnosis is established, HCC needs to be further evaluated by a staging system. The purpose of using malignant neoplasia staging systems is to eliminate ambiguity, thus contributing to the correct placement of patients in the appropriate therapeutic strategy; to estimate the prognosis; and to evaluate the response to treatment. The situation of patients with HCC is a particular one compared to other cancers, considering the presence of liver cirrhosis in most cases. The prognosis of patients depends on the stage of the two conditions, HCC and liver cirrhosis [40]. In order to overcome this shortcoming, numerous HCC staging systems have been proposed over the years, the most well-known being the BCLC (Barcelona Clinic Liver Cancer), Okuda, CLIP (Cancer of the Liver Italian Program), CUPI (Chinese University Prognostic Index), TNM, and JIS (Japan Integrated Staging) scores [41,42,43,44,45,46]. The most widely used classification is the BCLC classification.

## 4. Management of Hepatocellular Carcinoma Associated with NAFLD

The therapeutic management of HCC is complex and, according to the recommendations of current guidelines, it requires a multidisciplinary team consisting of hepatologists, oncologists, and surgeons specialized in liver surgery and transplantation, as well as radiologists. However, data from the literature show that only half of patients diagnosed with HCC are subsequently evaluated by a multidisciplinary team. Currently, the treatment recommendations for HCC are based on the BCLC classification and do not differ from one etiology to another, but do take into consideration the presence of liver cirrhosis and consequently liver function [41]. Placing patients in a specific therapeutic strategy depends on the BCLC classification, taking into account patient heterogeneity, patient wishes, ongoing clinical trials, and local limitations. There are scarce data regarding both treatment modalities and long-term survival in NAFLD-HCC, taking into consideration that these patients frequently have several comorbidities, such as type 2 diabetes mellitus, cardiovascular disease, and obesity. For instance, Wang et al. demonstrated that cirrhotic patients with type 2 diabetes and HCC have lower overall survival rates after curative hepatectomy compared to those without diabetes [47]. The authors concluded that diabetes may reduce the OS of HCC patients by exacerbating existing liver fibrosis, resulting in severe liver failure.

### 4.1. Hepatic Resection

In patients with HCC without liver cirrhosis and impaired liver function, hepatic resection represents the first option for treatment [30,31]. However, despite progress having been made in the last years in improving the survival rate in those with liver resection, the recurrence rate has not shown major changes. Research studies that assessed the overall survival (OS) and recurrence-free survival (RFS) in patients with NAFLD-associated HCC showed optimistic results (Table 1). It appears that OS at 5 years after liver resection for NAFLD-associated HCC ranges from 51.5% to 97%, whereas RFS at 5 years ranges from 36.3% to 66% [48,49,50,51,52,53,54]. However, there is an ongoing debate regarding the outcomes after resection in patients with NAFLD-associated HCC vs. other liver diseases. It appears that the presence of metabolic and cardiovascular comorbidities, which are often found in patients with NAFLD, has a negative impact on the OS after liver resection for HCC [55]. A meta-analysis that aimed to evaluate the outcome after hepatic resection for HCC in NAFLD vs. other liver diseases in approximately 7200 patients found a better RFS and OS in those with NAFLD [56]. Furthermore, a lower RFS was found in a study that compared NAFLD-associated HCC with HCV-related HCC (44.6% vs. 62.5%) [52]. Still, it is important to acknowledge that the high post-surgical mortality in patients with NAFLD is mainly due to the metabolic comorbidities, which should be carefully diagnosed and managed.

### 4.2. Ablation

Radiofrequency ablation (RFA) is a non-surgical treatment method that is currently recommended in patients with stage 0 (tumors smaller than 2 cm) or A, according to the BCLC classification, with OS rates similar to resection [41]. Regarding NAFLD-associated HCC, a recent study that evaluated the OS rates in patients treated with RFA for HCC in NAFD and other liver diseases reported no significant differences [50]. However, data from another study show that the presence of type 2 diabetes impairs the outcome after RFS, though metformin therapy has a positive impact on OS [57]. Despite the good efficacy and safety of microwave ablation of HCC, there are no data regarding the outcome in patients with NAFLD.

### 4.3. Liver Transplantation

According to the European Liver Transplant Registry, the survival rate at 10 years after liver transplantation for HCC is 51%, irrespective of underlying etiology [58]. The current guidelines recommend liver transplantation as the first choice in patients with HCC who do not meet the eligibility criteria for liver resection but are within the Milan criteria [30,31,32]. However, since many believe that the Milan criteria are too strict, nowadays there are many centers that offer liver transplantation in patients with HCC outside the Milan experience, with good results [59].

There are several studies regarding long-term outcomes after liver transplantation in NAFLD-associated HCC (Table 2). The OS and RFS rates range from 59% to 88% and 48% to 68%, respectively [49,60,61,62,63,64]. Although some studies reported similar outcomes after liver transplantation for HCC in NAFLD and other etiologies [49,65], there are some studies that raised concerns regarding worse OS in those with NAFLD. For instance, a comprehensive analysis from the European Transplant Registry, which included patients with liver transplantation for different etiologies, reported lower OS in NAFLD-HCC compared to alcoholic liver disease-related HCC [60]. On the other hand, the same authors found no difference in terms of OS rates at 10 years when compared to chronic HCV infection and cryptogenic cirrhosis (73%). In contrast with these results, an American study found no difference in OS rates after liver transplantation in NAFLD-associated HCC vs. alcoholic liver disease-associated HCC [49]. These differences could be attributed to different national listing and scoring systems. Overall, it seems that NAFLD has no significant impact on OS after liver transplantation for HCC compared to other causes of liver disease, but it needs to be taken into consideration due to the high complication rates after surgery due to metabolic syndrome-associated comorbidities.

Considering that nowadays there are numerous centers that offer liver transplantation beyond the Milan criteria, patients with NAFLD and HCC have also benefited from the extended indications. Rajendran et al. evaluated the outcomes of liver transplantation in patients with NAFLD-related HCC and found that there were no survival differences in populations within or beyond the Milan criteria [62].

### 4.4. Neoadjuvant and Adjuvant Therapies

Currently, there is no recommendation for adjuvant and neoadjuvant therapies use in HCC management because of the low efficacy and poor safety profile of the agents studied until now. Although HCC has very high rates of recurrence after resection or ablation (up to 70% at 5 years after curative treatment), there has been no therapy able to modify the outcome in these patients. There are several ongoing phase III randomized controlled trials that are evaluating the efficacy of adjuvant therapies after curative treatment with nivolumab, pembrolizumab, atezolizumab + bevacizumab, and durvalumab [66].

### 4.5. Transcatheter Arterial Chemoembolization

In patients with unresectable HCC and preserved liver function with no evidence of vascular invasion or extrahepatic spread, categorized as BCLC class B, the first-line treatment is transcatheter arterial chemoembolization (TACE). The classic method for TACE, consisting of the administration of an anticancer-in-oil emulsion followed by embolic agents, has been replaced in the last few years with a more efficient alternative that offers the possibility of introducing an embolic drug-eluting bead (DEB) providing a better efficacy and safety profile [67]. Data from several studies that assessed the pharmacokinetic profile of DEBs loaded with doxorubicin reported excellent features with lower systemic drug exposure and significantly reduced liver toxicity compared with conventional TACE [68,69,70].

In NAFLD-associated HCC, data about TACE efficacy are still scarce, with few studies mentioning its feasibility [71,72]. In a recent study, Young et al. retrospectively compared the median OS in patients with HCC and NAFLD vs. other etiologies after TACE and found that there were no significant differences [73]. In contrast with these results, another study conducted by Wu et al., which included 57 patients with HCC of different etiologies who had performed 100 TACE procedures, reported a negative impact of obesity on post-therapy residual disease and the time-to-progression interval [74]. Consequently, the low scientific evidence for TACE in NAFLD-associated HCC does not currently sustain a clear recommendation for including this procedure in the treatment strategy.

### 4.6. Systemic Therapy

Data regarding systemic therapy in NAFLD-associated HCC are lacking and the indication of treatment is derived from HCC cases of other etiologies.

The first agent for systemic therapy in HCC was sorafenib, which was introduced in 2007 based on the excellent results from the SHARP trial and has been used as a first-choice therapy for advanced-stage HCC (BCLC C) for over 10 years [74]. Data from the SHARP phase III trial showed that the efficacy of sorafenib varied depending on the etiology of HCC, being more effective in those with chronic HCV infection [75]. Interestingly, it has been recently demonstrated by a cohort study that included HCC patients with several etiologies of liver disease that the efficacy of sorafenib was similar in NAFLD patients when compared to other etiologies [76].

Recent advances in the field of immunotherapy for HCC have introduced new agents in the management of advanced-stage HCC, with promising results. The REFLECT trial demonstrated an improved OS of lenvatinib compared to sorafenib (13.6 vs. 12.3 months) [77]. Interestingly, lenvatinib showed an improvement of 1.5 months in terms of progression-free survival in patients with NAFLD-associated HCC compared to viral-related HCC [78]. Regorafenib has been recently recommended based on the improved survival rates in viral and non-viral HCC when compared to placebo (10.6 vs. 7.8 months), but due to the low incidence of NAFLD patients in the pivotal trial, there are no data regarding the efficacy of the drug in this cohort [79]. Similarly, the trials that evaluated cabozantinib and ramucirab, which, along with lenvatinib, are recommended as second-line choices when sorafenib fails, did not offer any data on their efficacy in NAFLD patients [80,81].

The cytotoxicity of these drugs is, however, a matter of concern nowadays. For instance, the side effects of antiangiogenic medications, such as sorafenib, may include hypertension, renal toxicity, arterial thromboembolism, bleeding, cardiotoxicity, thyroid dysfunction, hand–foot skin reaction, rash, pruritus, alopecia, potentially fatal hepatotoxicity, toxic/metabolic encephalopathy, and muscle wasting. On the other hand, despite having significantly higher transaminases than patients receiving immune checkpoint inhibitors for other conditions (such as lung cancer or melanoma), patients with HCC have not experienced early treatment termination or treatment-related mortality [82].

## 5. Remaining Challenges

As the pool of patients with viral-related HCC is decreasing worldwide due to efficient antiviral therapy and vaccination, NAFLD-associated HCC is gaining more attention. Thus, effective strategies for prevention and treatment are needed.

In terms of HCC surveillance, NAFLD patients have several features that could limit the detection of tumors. Firstly, there is a high percentage of patients with NAFLD-associated HCC who do not have liver cirrhosis and are not suitable for screening strategies [83]. A possible solution for this issue could be novel risk stratification algorithms for these patients. Secondly, the first-choice tool for screening—US—has been reported to miss early HCCs in NAFLD patients, mainly due to a decreased visualization attributed to obesity [84]. A cohort study which included 2053 patients with cirrhosis evaluated by US reported limited visualization (18.0% of patients), and this was independently associated with NAFLD (odds ratio (OR), 2.13; 95% CI, 1.51–3.00) or alcohol-associated cirrhosis (OR, 1.74; 95% CI, 1.25–2.43) and obesity class II (OR, 1.69; 95% CI, 1.06–2.67) or class III (OR, 4.35; 95% CI, 2.82–6.71) [85]. Considering this shortcoming, novel imaging and biomarker-based strategies are needed to increase the accuracy of early HCC detection. For instance, multi-biomarker panels, such as GALAD (gender, age, alpha-fetoprotein L3%, AFP, and des-gamma-carboxy prothrombin) scores, show very good performance, with 80% sensitivity for early HCC [86].

The goal of all therapy strategies is to increase efficacy while maintaining a good safety profile. Patients with NAFLD-associated HCC have a unique profile that is characterized by the presence of metabolic disorders, cardiovascular disease, and type 2 diabetes mellitus. These features could impair the outcome after surgery (resection and transplantation) or lower the efficacy of TACE and systemic therapy. Thus, the treatment strategy should be individualized based on the patient’s characteristics. Furthermore, it is important to acknowledge the high chance of developing HCC in non-cirrhotic patients with NAFLD, which is more frequent than in patients with viral or alcoholic etiologies, which implies that a more aggressive surveillance strategy is needed in order to diagnose early HCC. The question remains if such strategies are cost-efficient due to the low prevalence of HCC in non-cirrhotic NAFLD patients.

Regarding systemic therapies, there are scarce data about the efficacy and safety profile of local and systemic therapies due to the low proportion of NAFLD-associated HCC patients in the trials. Considering that most treatment modalities are influenced by the underlying etiologies, there is an urgent need for randomized controlled trials that are focused on these patients.

## 6. Conclusions

Due to the pandemic proportions that NAFLD has gained in the last few years, there is a high amount of data derived from basic and clinical studies that have revealed the molecular pathways that drive NAFLD-associated HCC. Based on these findings, new prevention, surveillance, and treatment strategies could be developed at the individual level. Current data on treatment modalities in NAFLD-associated HCC are still scarce, but based on subgroup analysis, the results from non-NAFLD HCC studies are promising and could provide a basis for a future research agenda to address NAFLD/NASH patients. Clinicians should carefully assess all the clinical and radiological parameters and establish a prognosis based on the BCLC classification and discuss in a multidisciplinary team the treatment strategy. The specific factors associated with NAFLD-associated HCC which can have a negative impact on survival even in patients with early HCC, such as cardiovascular disease, type 2 diabetes, and obesity, should be taken into consideration. Furthermore, the nomenclature and definition discord led to limitations which had a negative impact on clinical studies, but the recent change in the nomenclature of steatotic diseases aims to unify these patients under the right pathophysiological umbrella term, which will lead to improvement in study recruitment and clarify potential treatments and their applicability to this specific cohort, considering all their comorbidities.

## Figures and Tables

**Table 1:** Overall survival (OS) and recurrence-free survival (RFS) in patients with NAFLD-associated HCC after liver resection.

Ref.	Type of Study	Patients (n) and Characteristics	Overall Survival Rate *	Recurrence-Free Survival **
Koh et al. [33]	Retrospective	N = 996 HCC patients, 844 with non-NAFLD HCC and 152 with NAFLD HCC	70.1%	45.4%
Reddy et al. [34]	Retrospective	N = 214 HCC patients, 52 with NASH and 162 with HCV or ALD	59%	48%
Liang et al. [35]	Retrospective	N = 177 HCC patients, 75 with NASH and 102 with alcoholic or viral hepatitis	87%	51%
Vigano et al. [36]	Retrospective	N = 192 HCC patients, 96 with NASH and 96 with HCV	65.6%	37%
Billeter et al. [37]	Retrospective	N = 365 HCC patients, 62 with NASH and 303 with HCV	71.3%	36.3%
Yang et al. [38]	Retrospective	N = 1483 HCC patients, 96 with NAFLD HCC and 1387 with HBV HCC	51.4%	38.8%
Wakai et al. [39]	Retrospective	N = 225 HCC patients, 17 with NAFLD HCC, 61 with HBV, and 147 with HCV	59%	66%

* Five-year overall survival rate. ** Five-year recurrence free survival.

**Table 2:** Overall survival (OS) and recurrence-free survival (RFS) in patients with NAFLD-associated HCC after liver transplantation.

Ref.	Type of Study	Patients (n) and Characteristics	Overall Survival Rate *	Recurrence-Free Survival
Reddy et al. [34]	Retrospective	N = 214 HCC patients, 52 with NASH and 162 patients with HCV or ALD	59%	48% at 5 years
Haldar et al. [44]	Retrospective	N = 68,950 recipients, 1071 with NASH-HCC and 19,134 with HCC of other etiologies	68.6%	n/a
Wong C.R. et al. [45]	Retrospective	N = 17,644 HCC patients, 406 patients with NAFLD, 1854 with HCV, 1342 with HBV, and 1024 with ALD	60%	n/a
Rajendran et al. [46]	Retrospective	N = 20,672 HCC patients, 2071 with NASH HCC and 18,601 with HCC of other etiologies	76.3%	n/a
Sadler et al. [47]	Retrospective	N = 929 HCC patients, 60 with NASH and 869 with other etiologies	80%	68%
Malik et al. [48]	Retrospective	N = 17 NASH HCC patients	88% at 2.5 years	n/a

* Five-year overall survival rate.

## Data Availability

Not applicable.
